# Prediction of acute kidney injury incidence following acute type A aortic dissection surgery with novel biomarkers: a prospective observational study

**DOI:** 10.1186/s12916-023-03215-9

**Published:** 2023-12-18

**Authors:** Zhigang Wang, Jingfang Xu, Yu Zhang, Cheng Chen, Chuiyu Kong, Lu Tang, Yi Jiang, Ronghuang Yu, Qiuyan Zong, Lifang Zhang, Dongjin Wang

**Affiliations:** 1 Department of Cardio-Thoracic Surgery, Nanjing Drum Tower Hospital, Affiliated Hospital of Medical School, Nanjing University, Nanjing, China; 2 Department of Nephrology, Ningbo First Hospital, Ningbo, China; 3 Department of Pharmacology, College of Pharmacy, Harbin Medical University, Harbin, China; 4 Department of Cardiovascular Surgery, Nanjing Drum Tower Hospital, Chinese Academy of Medical Science & Peking Union Medical, Beijing, China; 5 Department of Psychiatry, The First Affiliated Hospital, Zhengzhou University, Zhengzhou, China

**Keywords:** Acute kidney injury, Type A aortic dissection, S100A8/A9, Pentraxin 3, Chitinase 3-like 1, Nomogram

## Abstract

**Background:**

Acute kidney injury (AKI) is a prevalent complication following acute type A aortic dissection (ATAAD) surgery and is closely associated with unfavorable prognostic outcomes. Hence, the development of a robust and efficient diagnostic approach to identify high-risk patients is of paramount importance.

**Methods:**

We conducted a prospective study involving 328 patients who underwent ATAAD surgery at our institution, comprising three distinct cohorts. In addition, 52 patients undergoing alternative cardiopulmonary surgeries and 37 healthy individuals were enrolled as control groups. Employing proteomic analysis, we initially identified plasma proteins potentially linked to AKI occurrence within the plasma proteomic cohort. Subsequent validation was performed in an independent cohort. Utilizing predictors derived from multivariate logistic regression analysis, a nomogram was meticulously formulated and its efficacy was validated in the model construction cohort.

**Results:**

Proteomics revealed significant elevation of plasma levels of S100A8/A9, pentraxin 3 (PTX3), and chitinase 3-like 1 (CHI3L1) immediately post-surgery in patients who developed ATAAD surgery-associated AKI (ASA-AKI). Receiver operating characteristic (ROC) curves demonstrated impressive predictive performance of S100A8/A9, PTX3, and CHI3L1 at 0 h post-surgery, yielding area under the curve (AUC) values of 0.823, 0.786, and 0.803, respectively, for ASA-AKI prediction. Furthermore, our findings exhibited positive correlations between plasma levels of S100A8/A9, PTX3, CHI3L1, and urinary neutrophil gelatinase-associated lipocalin (NGAL) at 0 h post-surgery, along with correlations between plasma S100A8/A9, CHI3L1 levels, and the Cleveland Clinic score. A logistic regression model incorporating plasma S100A8/A9, PTX3, CHI3L1 levels, urinary NGAL levels, and the Cleveland Clinic score facilitated the construction of a predictive nomogram for ASA-AKI. This nomogram demonstrated robust discriminative ability, achieving an AUC of 0.963 in the model construction cohort.

**Conclusions:**

Our study underscored the augmentation of plasma S100A8/A9, PTX3, and CHI3L1 levels immediately post-surgery in patients developing ASA-AKI. The incorporation of these three biomarkers, in conjunction with the Cleveland Clinic score and NGAL, into a nomogram demonstrated commendable predictive efficacy. This presents a practical tool for identifying patients at an elevated risk of AKI following ATAAD surgery.

**Supplementary Information:**

The online version contains supplementary material available at 10.1186/s12916-023-03215-9.

## Background

Acute type A aortic dissection (ATAAD) represents a critical condition within the realm of cardiovascular diseases, often necessitating emergent surgical intervention. Acute kidney injury (AKI) arising from cardiac surgery stands as the second most prevalent cause of AKI [1]. Research has demonstrated that the incidence of ATAAD surgery-associated AKI (ASA-AKI) is substantially higher in contrast to other cardiac surgeries [2], with rates ranging from 23 to 67% [3–6]. Notably, our previous investigation documented a 53.6% occurrence of ASA-AKI among patients undergoing surgery at our institution [7]. ASA-AKI has been established as an independent risk factor associated with short- and long-term mortality, prolonged stays in intensive care unit (ICU), extended hospitalization, and escalated healthcare expenditures [8, 9]. Regrettably, there remains an absence of effective therapies specifically targeting ASA-AKI [10]. Consequently, the timely identification of individuals at elevated risk is of pivotal significance to enable tailored management and timely interventions.

Traditionally, serum creatinine (sCr) has been employed as a diagnostic marker for AKI. However, sCr levels exhibit significant elevation only after a decline of over 50% in glomerular filtration rate, potentially missing the critical treatment window [11]. Cystatin C, a marker freely filtered through glomerular membranes, presents a more reliable indicator of glomerular filtration rate. Nevertheless, our prior study yielded an area under the receiver operating characteristic (ROC) curve of merely 0.687 for cystatin C in forecasting ASA-AKI [12]. Although recognized biomarkers like neutrophil gelatinase-associated lipocalin (NGAL) have been identified for cardiac surgery-associated AKI [13], there remains a void in dependable biomarkers for the prediction of ASA-AKI.

Over the last decade, proteomics has emerged as a robust tool to unearth disease-related proteins and novel biomarkers across various medical domains, encompassing acute interstitial nephritis and heart failure [14, 15]. However, proteomic investigations within ASA-AKI patients have remained relatively scarce. In this study, we harnessed plasma proteomics to delve into the expression profiles of plasma proteins following ATAAD surgery, pinpointing potential biomarkers correlated with ASA-AKI development. To forge an encompassing predictive nomogram, we amalgamated these biomarkers with the Cleveland Clinic score—an extensively adopted risk assessment model for postoperative adverse renal events [16]. We aspire for this predictive model to consistently and accurately discern high-risk patients predisposed to ASA-AKI, thereby advancing clinical management.

## Methods

### Study population

This prospective observational study unfolded across three successive cohorts. Perioperative plasma samples and pertinent clinical data were meticulously collated from a consecutive stream of ATAAD-diagnosed patients at Nanjing Drum Tower Hospital, spanning from June 1, 2021, to March 31, 2023. The study inclusion criteria encompassed patients diagnosed with ATAAD via computed tomography angiography (CTA), having undergone surgery within five days of ATAAD onset. Exclusions were extended to encompass patients with preoperative end-stage renal failure, preoperative renal malperfusion, or those succumbing within 48 h post-surgery. Instances of missing plasma samples were similarly omitted. From an initial screening pool of 371 patients, a final cohort of 328 was established and subdivided into three distinct groups: the plasma proteomic cohort (comprising 15 ASA-AKI and 15 non-ASA-AKI patients), the validation cohort (comprising 76 eligible patients), and the model construction cohort (comprising 222 eligible patients).

Concurrently, plasma S100A8/A9, pentraxin 3 (PTX3), and chitinase 3-like 1 (CHI3L1) levels were gauged and juxtaposed against those of 37 age-, gender-, and body mass index- (BMI-) matched healthy individuals. An additional independent set, consisting of 52 patients (equivalently age-, gender-, and BMI-matched), who had undergone cardiopulmonary (CPB) surgery, was enlisted to assess the specificity of plasma proteins in the context of ATAAD.

The incidence of AKI consistently served as the primary outcome throughout the study. The follow-up period remained consistent across all phases of the research, and the sample method was consistently upheld, ensuring uniformity in participant selection. Our emphasis was on maintaining a high level of methodological consistency to guarantee the reliability and validity of our study findings. The acquisition of biological samples adhered to a protocol endorsed by the Institutional Research Ethics Committee of Nanjing Drum Tower Hospital (Approval Number: 2021–084-01), accompanied by informed consent secured from all participating patients.

### Definitions

The diagnostic criteria for AKI were grounded in the Kidney Disease Improving Global Outcomes (KDIGO) guidelines, with assessments conducted within seven days following surgery [17]. The baseline sCr or initial sCr for patients with ATAAD is typically assessed just before the surgical procedure after admission to the ICU. Patient medical records were carefully assessed to validate the diagnosis of chronic kidney disease (CKD) and determine the respective CKD stages. Malperfusion in the context of ATAAD was defined as a decrement in blood flow or complete artery occlusion, substantiated through preoperative CTA and corroborated by clinical indicators such as coma, extremity paralysis, or abdominal pain, along with pertinent laboratory results including elevated myocardial enzymes. The analysis of all CTA images was undertaken independently by two experienced radiologists. Preoperative hypotension was defined by mean arterial pressure falling below 75 mmHg.

### Sample collection, preservation, and measurement procedures

Routine laboratory samples, collected via an indwelling venous cannula pre-operatively in the ICU, were continued immediately after surgery and daily up to post-operative day 7 for sCr measurements. These samples were diligently sent for analysis to the hospital's clinical laboratory department. In cases where patients were diagnosed with AKI, plasma samples were extracted almost daily throughout their hospitalization period. This frequent sampling aimed to capture the dynamic fluctuations in sCr levels, enabling a comprehensive understanding of its behavior.

Measurement of S100A8/A9, PTX3, CHI3L1, and NGAL levels was undertaken to employ commercial enzyme-linked immunosorbent assay (ELISA) kits (ABclonal, Wuhan, China) within a span of 3 months from sample collection, in accordance with the manufacturer's instructions. The laboratory analyses were meticulously conducted in a blinded manner, ensuring complete separation from both the associated clinical data and the KDIGO classification. Upon collection, plasma and urinary samples were promptly centrifuged (3000 rpm for 30 min) or refrigerated at 4 °C and subsequently centrifuged within 4 h. Serum and urine supernatants were meticulously collected into EP tubes and expeditiously stored at − 80 °C for subsequent utilization. The limitation of repeated freeze–thaw cycles was meticulously adhered to. Plasma S100A8/A9 was assessed with a 5.0% intra-assay coefficient of variation (CV) and a 7.2% inter-assay CV, establishing a lower detection limit of 3.0 μg/L. PTX3 measurements demonstrated a precision with a 4.1% intra-assay CV and a 4.3% inter-assay CV, highlighting a remarkable lower detection limit of 0.1 ng/mL. Serum concentrations of CHI3L1 were determined with a lower detection limit of 20 ng/mL, displaying an intra-assay CV of 5.0% and an inter-assay CV of 5.4%. In the case of urinary NGAL tests, the lower detection limit was established at 10 ng/mL, while both intra- and inter-assay CVs ranged between 5 and 10% for batched samples analyzed on the same day.

**Fig. 1:**
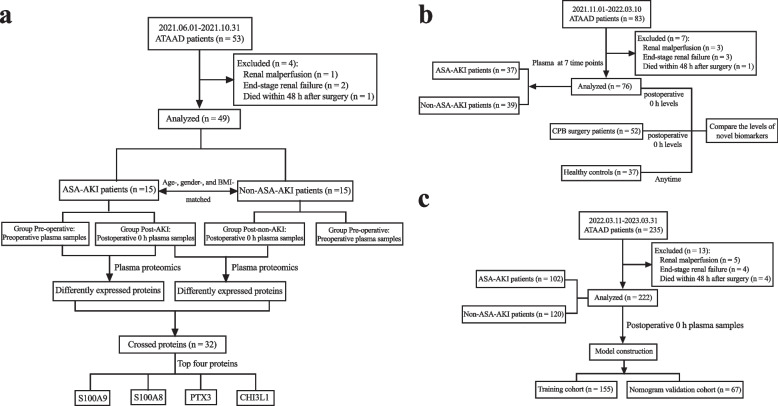
The study flowchart. **a** Enrollment and exclusion of patients in the ATAAD cohort, resulting in the selection of 15 patients with ASA-AKI and 15 age-, gender-, and BMI-matched non-ASA-AKI patients for proteomics analysis. **b** Composition of the validation cohort, comprising 76 ATAAD patients after exclusion of 7 patients. **c** Model construction cohort, with 235 ATAAD patients screened and 222 patients enrolled for further analysis. ATAAD, acute type A aortic dissection; BMI, body mass index; ASA-AKI, acute type A aortic dissection surgery-associated acute kidney injury

The plasma proteomic cohort consisted of 15 ASA-AKI and 15 non-ASA-AKI patients, matched for age, gender, and BMI, encompassing the period from June 1, 2021, to October 31, 2021. The quantification of protein expression, facilitated through isobaric tags for relative and absolute quantification labeling coupled with liquid chromatography-tandem mass spectrometry analysis, was executed by Applied Protein Technology (Shanghai, China), aligning with established protocols [18]. The schematic depiction of case inclusion and proteomic measurements is presented in Fig. 1a.

Validation cohort: the validation cohort, comprised of 76 eligible patients, was enrolled between November 1, 2021, and March 10, 2022. Blood samples were diligently collected at seven distinct time points: preoperative, postoperative 0 h, postoperative 6 h, postoperative 12 h, postoperative 24 h, postoperative 48 h, and postoperative 72 h. The comprehensive depiction of case inclusions is presented in Fig. 1b.

Healthy and disease control cohort: a total of 37 healthy volunteers, including 27 males and 10 females (with ages ranging from 40 to 72 years; mean age of 55.8 ± 8.2 years), who sought annual physical examinations at Nanjing Drum Tower Hospital between January 1, 2022, and January 1, 2023, were encompassed in this study. A meticulous assessment, inclusive of consultation, physical examination, blood pressure measurements, specialized examinations, and scrutiny of medical records, was conducted for each volunteer. Fasting blood samples were collected from all volunteers on a single occasion. In parallel, an additional group of 52 patients, who had undergone CPB surgery at our institution between January 1, 2022, and January 1, 2023, were enrolled as a disease control cohort. This subgroup consisted of 28 patients who underwent isolated valve replacement surgery and 24 patients who underwent isolated coronary artery bypass grafting surgery. Blood samples were obtained from these patients both preoperatively and immediately postoperative.

Model Construction Cohort: the model construction cohort, consisting of 222 eligible patients, was enrolled between March 11, 2022, and March 31, 2023. Blood samples were meticulously collected at 0 h post-surgery. The comprehensive depiction of case inclusions for this cohort is depicted in Fig. 1c.

### Statistical analysis

Statistical analyses were meticulously conducted utilizing SPSS 26.0 (IBM, Chicago, IL, USA). Continuous variables underwent comparison through the Student's *t*-test or the Mann–Whitney *U* test, with representation as mean ± standard deviation for normally distributed data, and as median along with interquartile range for non-normally distributed data. The chi-square test or Fisher’s exact test was employed for the comparison of categorical data, presented as counts and percentages. Spearman's correlation analysis was executed to explore associations between serum levels of S100A8/A9, PTX3, and CHI3L1 at 0 h post-surgery and diverse variables. The diagnostic efficiency of the biomarkers was assessed through the construction of ROC curves and the calculation of the corresponding area under the curve (AUC) values.

Sample size determination was carried out, indicating a need for approximately 194 patients to achieve 95% statistical power in detecting a clinically meaningful ASA-AKI rate of 45.0% at a two-sided α of 0.05. Proteomics were plotted and analyzed using the R program (version 4.1.3), with Volcano plots generated utilizing the ggplot2 package. Statistically significant differences were attributed to a protein fold change exceeding 2 or falling below 0.5 (*p* < 0.05). The predictive model was constructed and visualized employing the “rms” package under the R language platform. In summary, the patient cohort was randomly partitioned into training and validation groups at a 7:3 ratio. The associations between each preoperative variable and the occurrence of ASA-AKI were evaluated by Spearman’s correlation analysis. Univariate analysis and multivariate analysis were performed to identify risk factors for ASA-AKI. The selection of the final subset of variables was carried out through the forward, backward, or both elimination model selection procedures, utilizing the Wald test as a basis for assessment. To determine the most appropriate model fit, metrics such as deviance, Akaike information criterion (AIC), Bayesian information criterion (BIC), and pseudo-R^2^ were employed, indicating the robustness and suitability of the model statistics. In establishing a risk scoring model, a range of identified factors was considered. These factors included demographic characteristics, comorbidities, preoperative laboratory values, preoperative radiologic characteristics, operative data, and postoperative variables. Their comprehensive inclusion allowed for a holistic approach, capturing various aspects that could contribute to the assessment and prediction of risk in the given context.

The constructed nomogram underwent validation through 1000 bootstrap resamples. The AUC of the ROC curve and precision-recall curve were used to evaluate the discrimination ability of the nomogram. Calibration curve analysis was conducted to assess the nomogram's calibration proficiency. Decision curve analysis was further undertaken to ascertain the practical applicability of the nomogram within the validation cohort. All analyses adhered to a two-sided principle, with *p*-values less than 0.05 deemed statistically significant.

## Results

### Distinct protein expression profiles identified in ASA-AKI patients via proteomics

Plasma samples collected before and immediately after completion of ATAAD surgery were subjected to proteomic analysis. As indicated in Fig. 1a, 53 patients underwent ATAAD surgical repair between June 1, 2021, and October 31, 2021. Following the exclusion of four patients (1 due to renal malperfusion, 2 with end-stage renal failure, and 1 deceased within 48 h after surgery), 49 patients were included in the subsequent analysis. Among these, 24 patients (49.0%) developed AKI post-surgery, comprising 8 patients in KDIGO stage 1, 9 patients in KDIGO stage 2, and 7 patients in KDIGO stage 3. After carefully matching for age, gender, and BMI, samples from 15 patients who developed ASA-AKI and 15 patients who did not develop ASA-AKI were sent for further proteomic analysis. Characteristics of these 30 patients were outlined in Table 1, revealing no significant differences in preoperative variables between the groups. However, notable differences were observed in intraoperative parameters, with prolonged CPB duration (206.3 ± 49.2 min vs. 166.3 ± 35.5 min; *p* = 0.016) and cross-clamp duration (155.8 ± 47.3 min vs. 125.1 ± 33.0 min; *p* = 0.048) in the ASA-AKI group.

**Table 1:** Demographic and clinical data of acute type A aortic dissection patients whose samples were sent for plasma proteomics

Variables	Overall (*n* = 30)	ASA-AKI (*n* = 15)	Non-ASA-AKI (*n* = 15)	*p*-value
***Preoperative parameters***				
Demographics				
Age (year)	63.3 ± 14.3	62.6 ± 13.3	64.1 ± 15.6	0.784
Male (%)	21 (70.0)	10 (66.7)	11 (73.3)	0.690
BMI (kg/m^2^)	23.9 ± 3.4	24.6 ± 3.0	23.3 ± 3.7	0.289
Tobacco use (%)	11 (36.7)	6 (40.0)	5 (33.3)	0.705
Medical history				
Hypertension (%)	27 (90.0)	13 (86.7)	14 (93.3)	1.000
Diabetes mellitus (%)	2 (6.7)	1 (6.7)	1 (6.7)	1.000
Previous cardiac surgery (%)	2 (6.7)	1 (6.7)	1 (6.7)	1.000
Cerebrovascular disease (%)	7 (23.3)	3 (20.0)	4 (26.7)	1.000
Coronary artery disease (%)	1 (3.3)	0 (0)	1 (6.7)	1.000
CKD (%)	3 (10.0)	2 (13.3)	1 (6.7)	1.000
CKD stage 1 (%)	2 (6.7)	1 (6.7)	1 (6.7)	1.000
CKD stage 2 (%)	1 (3.3)	1 (6.7)	0 (0)	1.000
LVEF (%)	57.0 ± 3.0	57.3 ± 3.0	56.7 ± 3.1	0.592
Involving renal artery (%)	19 (63.3)	10 (66.7)	9 (60.0)	0.705
Left renal artery				
True lumen (%)	17 (56.7)	7 (46.7)	10 (66.7)	
False lumen (%)	5 (16.7)	3 (20.0)	2 (13.3)	
True and false lumen (%)	8 (26.7)	5 (33.3)	3 (20.0)	0.605
Right renal artery				
True lumen (%)	19 (63.3)	8 (53.3)	11 (73.3)	
False lumen (%)	5 (16.7)	2 (13.3)	3 (20.0)	
True and false lumen (%)	6 (20.0)	5 (33.3)	1 (6.7)	0.250
Preoperative laboratory results				
WBC (10^9^/L)	10.5 ± 3.2	10.1 ± 3.3	10.9 ± 3.2	0.543
Hemoglobin (g/L)	125.0 ± 23.6	120.9 ± 26.2	129.1 ± 20.7	0.346
Creatinine (μmol/L)	69.9 ± 20.7	72.7 ± 22.9	67.0 ± 18.7	0.479
BUN (mmol/L)	5.7 ± 1.8	5.5 ± 1.9	5.9 ± 1.8	0.583
Cystatin C (mg/L)	0.8 ± 0.4	0.9 ± 0.5	0.8 ± 0.4	0.625
eGFR (ml/min)	106.8 ± 20.1	104.0 ± 19.1	109.6 ± 21.3	0.454
Albuminuria (%)	2 (6.7)	1 (6.7)	1 (6.7)	1.000
D-Dimer (ng/mL)	8.1 (2.6, 23.1)	7.0 (2.4, 20.8)	10.2 (6.5, 39.0)	0.382
***Intraoperative parameters***				
CPB duration (min)	186.3 ± 46.8	206.3 ± 49.2	166.3 ± 35.5	0.016
Cross-clamp duration (min)	140.4 ± 43.0	155.8 ± 47.3	125.1 ± 33.0	0.048
Hypothermia circulation arrest time (min)	25.2 ± 9.8	24.5 ± 7.4	25.9 ± 12.0	0.704
***Postoperative parameters***				
Drainage volume 24 h after surgery (ml)	662.0 ± 278.9	775.3 ± 257.7	548.7 ± 259.1	0.023
Dialysis (%)	3 (10.0)	3 (20.0)	0 (0)	0.224
Cleveland Clinic score	4.3 ± 1.6	5.1 ± 1.6	3.5 ± 1.3	0.006
Mechanical ventilation time (h)	20.8 (16.0, 34.6)	34.5 (17.0, 76.5)	15.5 (15.0, 26.0)	0.032
In-hospital death (%)	3 (10.0)	2 (13.3)	1 (6.7)	1.000
ICU stay (days)	5.0 (3.0, 8.3)	5.0 (4.0, 9.0)	4.0 (3.0, 6.0)	0.005
Hospital stay (days)	17.0 (13.0, 22.3)	17.0 (14.0, 24.0)	15.0 (11.0, 18.0)	0.004

*BMI* Body mass index, *LVEF* Left ventricular ejection fraction, *WBC* White blood cell, *BUN* Blood urea nitrogen, *eGFR* estimated glomerular filtration rate, *CKD* Chronic kidney disease, *CPB* Cardiopulmonary bypass, *ICU* Intensive care unit

**Fig. 2:**
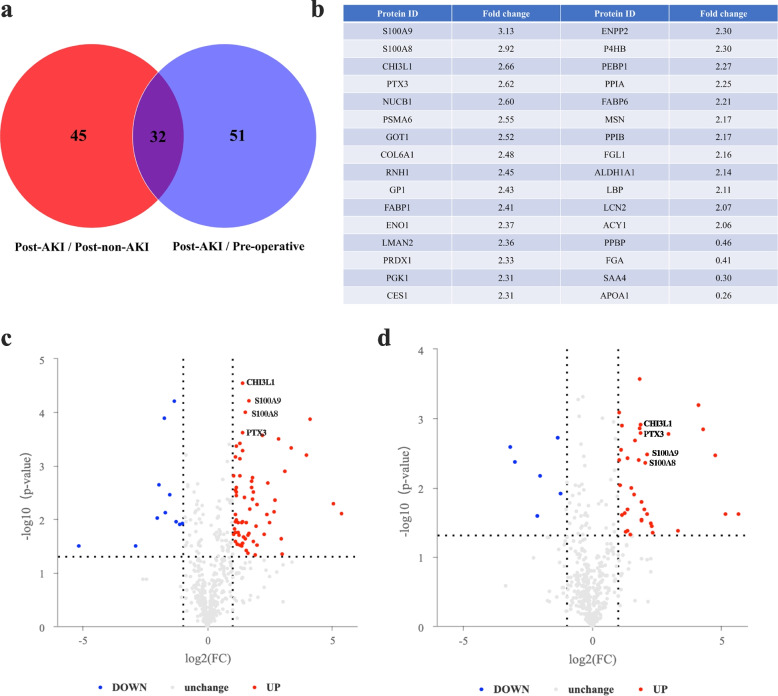
Screening of plasma biomarkers in ASA-AKI patients by proteomics. **a** Venn diagram depicting the number of differentially expressed proteins between the post-AKI group (0 h after surgery) vs. post-non-AKI group (0 h after surgery), as well as the post-AKI (0 h after surgery) group vs. pre-operative group (preoperative timepoint), with 32 crossed proteins identified. **b** List of the crossed proteins and their corresponding fold changes. **c**–**d** Volcano plots illustrating the up-regulation of S100A8, S100A9, PTX3, and CHI3L1 in ASA-AKI patients compared to non-ASA-AKI patients at postoperative 0 h (**c**), and preoperative measurements (**d**). ASA-AKI, acute type A aortic dissection surgery-associated acute kidney injury; PTX3, pentraxin 3; CHI3L1, chitinase 3-like 1

The list of proteins exhibiting differential expression was visualized in Fig. 2a (*p* < 0.05). A Venn diagram highlighted 77 proteins displaying postoperative differential expression between ASA-AKI and non-ASA-AKI patients. Additionally, 83 differentially expressed proteins were identified in ASA-AKI patients compared to preoperative measurements. Among these, 28 proteins were up-regulated and 4 were down-regulated postoperatively in ASA-AKI patients compared to both the non-ASA-AKI and preoperative groups. Figure 2b–c illustrate that S100A9 exhibited the highest fold change (3.13-fold) postoperatively in ASA-AKI patients compared to non-ASA-AKI patients, followed by S100A8 (2.92-fold), CHI3L1 (2.66-fold), and PTX3 (2.62-fold). These four proteins also displayed up-regulation postoperatively compared to preoperative measurements in ASA-AKI patients (Fig. 2d). Notably, S100A8 and S100A9 primarily exist as biologically functional S100A8/A9 heterodimers [19], rendering them subjects of further analysis.

### Validation of S100A8/A9, PTX3, and CHI3L1 levels via ELISA

**Fig. 3:**
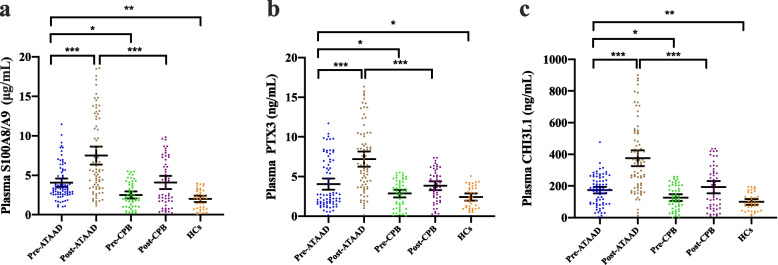
Plasma levels of S100A8/A9, PTX3, and CHI3L1 in healthy subjects and patient samples. **a**–**c** ELISA results demonstrating S100A8/A9 (**a**), PTX3 (**b**), and CHI3L1 (**c**) expression levels in plasma samples collected from ATAAD patients preoperatively (pre-ATAAD), postoperatively (post-ATAAD), disease controls preoperatively (pre-CPB), disease controls postoperatively (post-CPB), and healthy controls (HCs). ATAAD, acute type A aortic dissection; CPB, cardiopulmonary bypass; HCs, healthy controls; PTX3, pentraxin 3; CHI3L1, chitinase 3-like 1. Data were represented as mean ± SD, *p < 0.05; **p < 0.01; ***p < 0.001

To validate proteomics findings, S100A8/A9, PTX3, and CHI3L1 levels were measured using ELISA in the validation cohort, and compared with healthy and disease-control cohorts. Figure 1b outlines the enrollment of 76 ATAAD patients, 37 age-, gender-, and BMI-matched healthy individuals, and 52 age-, gender-, and BMI-matched patients who underwent CPB surgery for comparison. Among ATAAD patients, a collective total of 37 individuals, representing 48.7% of the cases, experienced postoperative AKI. This group comprised 13 patients classified under KDIGO stage 1, 12 patients under KDIGO stage 2, and 12 patients under KDIGO stage 3. Clinical and laboratory characteristics of enrolled patients are presented in Additional file 1: Table S1 and S2. The data revealed prolonged CPB duration and cross-clamp duration in the ASA-AKI group compared to the non-ASA-AKI group within the ATAAD cohort (Additional file 1: Table S1) and between the ATAAD and disease control groups (Additional file 1: Table S2). As illustrated in Fig. 3a–c, preoperative plasma levels of S100A8/A9, PTX3, and CHI3L1 were significantly elevated in ATAAD patients compared to preoperative levels of patients undergoing CPB surgery, as well as healthy controls. Postoperative levels of S100A8/A9, PTX3, and CHI3L1 were markedly higher in ATAAD patients compared to the disease control group.

**Fig. 4:**
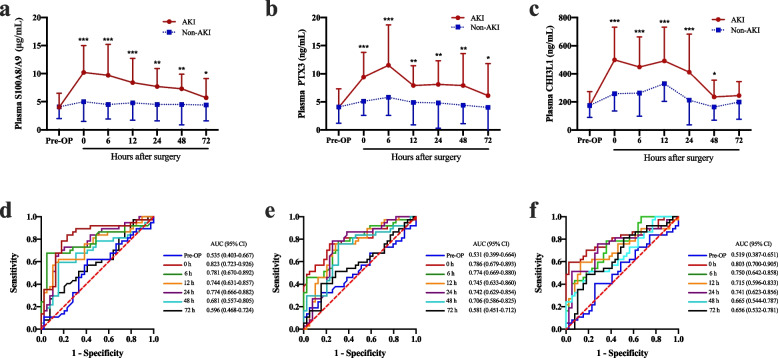
Dynamic changes of S100A8/A9, PTX3, and CHI3L1 and the AUC results in patients who developed ASA-AKI. **a-c** Plasma levels of S100A8/A9 (**a**), PTX3 (**b**), and CHI3L1 (**c**) in ASA-AKI and non-ASA-AKI patients at different time points. **d-f** ROC curves of plasma S100A8/A9 (**d**), PTX3 (**e**), and CHI3L1 (**f**) to detect ASA-AKI at different time points. ASA-AKI, acute type A aortic dissection surgery-associated acute kidney injury; PTX3, pentraxin 3; CHI3L1, chitinase 3-like 1; ROC, receiver operating characteristic; AUC, area under the curve; CI, confidence interval. Data were represented as mean ± SD, *p < 0.05; **p < 0.01; ***p < 0.001

Dynamic changes of these biomarkers in ASA-AKI patients were depicted in Fig. 4a–c. S100A8/A9 and CHI3L1 levels peaked at 0 h after surgery, whereas PTX3 levels peaked at 6 h post-surgery, followed by a subsequent decline in ASA-AKI patients. In contrast, patients without ASA-AKI experienced slight postoperative increases in these proteins compared to preoperative levels. Importantly, plasma levels of these biomarkers at various time points post-surgery were significantly higher in the ASA-AKI group compared to the non-ASA-AKI group. Evaluation of diagnostic value indicated that AUCs of S100A8/A9 at 0 h, 6 h, 12 h, and 24 h post-surgery were 0.823, 0.781, 0.744, and 0.774, respectively (Fig. 4d). The AUCs for PTX3 at corresponding time points were 0.786, 0.774, 0.745, and 0.742 (Fig. 4e), while those for CHI3L1 were 0.803, 0.750, 0.715, and 0.741 (Fig. 4f). The urinary levels of NGAL exhibited a gradual increase post-surgery, with a notable difference observed in the early postoperative period between ASA-AKI patients and non-ASA-AKI patients (Additional file 1: Fig. S1a). The diagnostic assessment revealed that the AUCs of urinary NGAL at 0 h and 24 h post-surgery were 0.633 and 0.605, respectively (Additional file 1: Fig. S1b). These findings suggest the potential utility of these plasma proteins as diagnostic markers for ASA-AKI.

### Associations between plasma protein levels and other variables

Correlations between plasma levels of S100A8/A9, PTX3, CHI3L1, and their urinary expressions were explored. Positive correlations were observed between plasma levels of S100A8/A9 (*r* = 0.634, *p* < 0.001; Additional file 1: Fig. S2a) and PTX3 (*r* = 0.380, *p* < 0.001; Additional file 1: Fig. S2b) with their corresponding urinary levels at 0 h post-surgery, whereas no significant correlation was noted between CHI3L1 plasma level and its urinary counterpart (*r* = 0.163, *p* = 0.160; Additional file 1: Fig. S2c). Urinary levels of these proteins peaked at 24 h post-surgery and showed a significant difference in the ASA-AKI group compared to the non-ASA-AKI group at 24 h and 48 h post-surgery (Additional file 1: Fig. S2d–f). Urinary levels of S100A8/A9, PTX3, and CHI3L1 at 24 h post-surgery exhibited superior predictive value (AUCs: 0.770, 0.742, and 0.624, respectively) compared to other time points (Additional file 1: Fig. S2g–i). Notably, their predictive value in diagnosing ASA-AKI appeared less optimal than their plasma levels.

Associations were investigated between plasma levels of S100A8/A9, PTX3, CHI3L1, and urinary NGAL levels at 0 h post-surgery, as well as Cleveland Clinic scores. As listed in Additional file 1: Fig. S3a and Fig. S3d, plasma levels of S100A8/A9 correlated positively with urinary NGAL levels (*r* = 0.286,* p* = 0.012) and Cleveland Clinic scores (*r* = 0.394, *p* < 0.001). Similarly, plasma levels of CHI3L1 exhibited positive correlations with urinary NGAL levels (*r* = 0.325, *p* = 0.004; Additional file 1: Fig. S3c) and Cleveland Clinic scores (*r* = 0.245, *p* = 0.033; Additional file 1: Fig. S3f). Conversely, plasma levels of PTX3 correlated solely with urinary NGAL levels (*r* = 0.311, *p* = 0.006; Additional file 1: Fig. S3b), lacking correlation with Cleveland Clinic scores (*r* = 0.016, *p* = 0.891; Additional file 1: Fig. S3e). ROC analyses indicated better predictive values of S100A8/A9, PTX3, and CHI3L1 plasma levels at 0 h post-surgery compared to urinary NGAL levels and Cleveland Clinic scores (Additional file 1: Fig. S3g).

### Development of a nomogram for ASA-AKI prediction

The model construction cohort encompassed 222 patients (Fig. 1c). Detailed demographic and clinical characteristics were outlined in Table 2. A notable 45.9% of patients developed postoperative AKI, with 8.1% requiring renal replacement therapy. A total of 37 patients were categorized in KDIGO stage 1, 35 patients in KDIGO stage 2, and 30 patients in KDIGO stage 3. Hospital mortality reached 8.6%, climbing to 15.7% in patients with postoperative AKI. The cohort was randomly divided into training (155 patients) and nomogram validation (67 patients) cohorts. No significant differences were observed in perioperative variables between the two groups, except for the prevalence of diabetes mellitus (Additional file 1: Table S3).

**Table 2:** Demographic and clinical characteristics of the model construction cohort

Variables	Overall (*n* = 222)	ASA-AKI (*n* = 102)	Non-ASA-AKI (*n* = 120)	*p*-value
***Preoperative parameters***				
Demographics				
Age (year)	53.0 ± 13.6	53.6 ± 15.1	52.5 ± 12.2	0.565
Male (%)	172 (77.5)	77 (75.5)	95 (79.2)	0.513
BMI (kg/m^2^)	26.2 ± 4.6	26.6 ± 5.1	25.9 ± 4.1	0.266
Medical history				
Hypertension (%)	178 (80.2)	81 (79.4)	97 (80.8)	0.791
Diabetes mellitus (%)	8 (3.6)	4 (3.9)	4 (3.3)	1.000
Previous cardiac surgery (%)	10 (4.5)	3 (2.9)	7 (5.8)	0.349
Cerebrovascular disease (%)	25 (11.3)	11 (10.8)	14 (11.7)	1.000
CKD (%)	17 (7.7)	15 (14.7)	2 (1.7)	< 0.001
CKD stage 1 (%)	5 (2.3)	4 (3.9)	1 (0.8)	0.183
CKD stage 2 (%)	9 (4.1)	8 (7.8)	1 (0.8)	0.013
CKD stage 3 (%)	3 (1.4)	3 (2.9)	0 (0)	0.095
Involving renal artery (%)	150 (67.6)	69 (67.6)	81 (67.5)	0.981
Limb ischemia (%)	16 (7.2)	7 (6.9)	9 (7.5)	1.000
Mesenteric ischemia (%)	7 (3.2)	4 (3.9)	3 (2.5)	0.706
Cerebral ischemia (%)	13 (5.9)	5 (4.9)	8 (6.7)	0.577
Coronary ischemia (%)	9 (4.1)	5 (4.9)	4 (3.3)	0.736
Hypotension (%)	5 (2.3)	2 (2.0)	3 (2.5)	1.000
Preoperative laboratory results				
WBC (10^9^/L)	12.0 ± 3.9	13.2 ± 4.2	11.0 ± 3.4	< 0.001
Hemoglobin (g/L)	129.9 ± 20.1	129.8 ± 18.5	130.0 ± 21.5	0.929
Creatinine (μmol/L)	93.4 ± 49.2	105.0 ± 62.3	83.5 ± 31.1	0.002
BUN (mmol/L)	7.1 ± 2.8	7.4 ± 3.0	6.7 ± 2.5	0.061
Cystatin C (mg/L)	1.0 ± 0.4	1.1 ± 0.3	0.9 ± 0.4	< 0.001
eGFR (ml/min)	95.1 ± 36.4	89.4 ± 40.2	100.0 ± 32.2	0.033
Albuminuria (%)	8 (3.6)	7 (6.9)	1 (0.8)	0.025
D-dimer (ng/mL)	7.0 (3.8, 14.7)	6.3 (3.8, 15.1)	7.7 (4.1, 14.7)	0.428
***Intraoperative parameters***				
CPB duration (min)	186.5 (161.0, 224.0)	192.0 (164.8, 227.0)	183.5 (156.5, 220.8)	< 0.001
Cross-clamp time (min)	135.0 (113.8, 172.0)	140.0 (114.0, 172.8)	133.0 (112.3, 171.5)	< 0.001
Hypothermia circulation arrest time (min)	24.9 ± 10.9	24.7 ± 11.1	25.1 ± 10.7	0.789
***Postoperative parameters***				
Drainage volume 24 h after surgery (ml)	550.4 ± 457.8	622.9 ± 563.5	488.9 ± 334.0	0.038
Dialysis (%)	18 (8.1)	18 (17.6)	0 (0)	< 0.001
Cleveland Clinic score	5.4 ± 2.5	6.2 ± 2.6	4.8 ± 2.1	< 0.001
Mechanical ventilation time (h)	20.8 (14.0, 83.5)	48.0 (15.8, 112.5)	18.0 (13.0, 45.0)	< 0.001
In-hospital death (%)	19 (8.6)	16 (15.7)	3 (2.5)	0.001
ICU stay (days)	5.0 (3.0, 8.0)	6.0 (4.0, 11.3)	4.0 (3.0, 6.0)	< 0.001
Hospital stay (days)	18.6 ± 10.4	19.9 ± 11.8	17.4 ± 9.0	0.037

*BMI* Body mass index, *WBC* White blood cell, *BUN* Blood urea nitrogen, *eGFR* estimated glomerular filtration rate, *CKD* Chronic kidney disease, *CPB* Cardiopulmonary bypass, *ICU* Intensive care unit

**Fig. 5:**
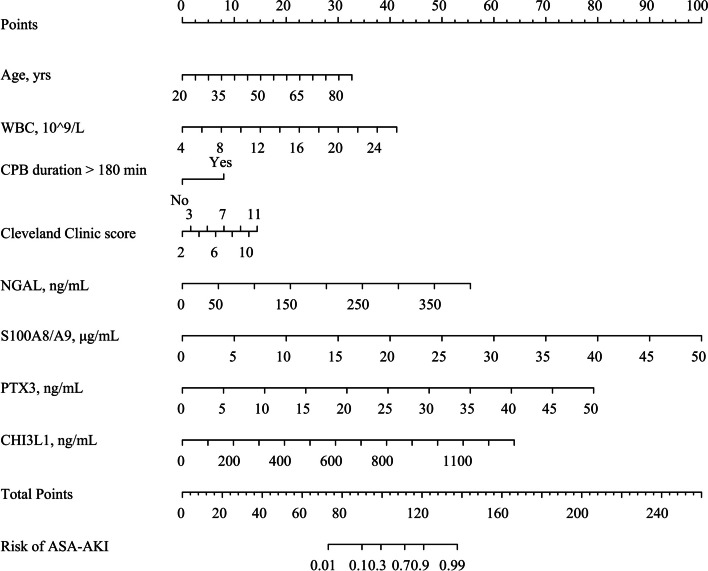
Nomogram for ASA-AKI. Scores assigned to different variables in the nomogram determine the risk of developing ASA-AKI. WBC, white blood cell; CPB, cardiopulmonary bypass; NGAL, neutrophil gelatinase-associated lipocalin; PTX3, pentraxin 3; CHI3L1, chitinase 3-like 1; ASA-AKI, acute type A aortic dissection surgery-associated acute kidney injury

The univariate logistic regression analysis identified potential risk factors for ASA-AKI, incorporating various factors such as age, BMI, pre-admission CKD status, preoperative estimated glomerular filtration rate, preoperative cystatin C, preoperative white blood cell count (WBC), preoperative sCr, preoperative D-dimer, duration of CPB exceeding 180 min, postoperative drainage volume 24 h after surgery, Cleveland Clinic score, and biomarkers levels (0 h post-surgery), encompassing NGAL, S100A8/A9, PTX3, and CHI3L1. Subsequently, the forward elimination model revealed specific influential factors, including age (odds ratio [OR] = 1.07, 95% confidence interval [CI]: 1.02–1.13, *p* = 0.005), WBC (OR = 1.31, 95% CI: 1.11–1.56, *p* = 0.002), CPB duration > 180 min (OR = 3.10, 95% CI: 1.13–8.96,* p* = 0.031), Cleveland Clinic score (OR = 1.26, 95% CI: 1.02–1.58, *p* = 0.037), NGAL (OR = 1.02, 95% CI: 1.01–1.03,* p* < 0.001), S100A8/A9 (OR = 1.33, 95% CI: 1.18–1.54, *p* < 0.001), PTX3 (OR = 1.25, 95% CI: 1.13–1.41, *p* < 0.001), and CHI3L1 (OR = 1.01, 95% CI: 1.00–1.01, *p* < 0.001). These factors displayed the minimal deviance, AIC, BIC, and the maximum pseudo-R^2^, signifying the best goodness of fit (Table 3 and Additional file 1: Table S4). The coefficients identified were used to construct a nomogram for assessing the risk of ASA-AKI (Fig. 5), offering a practical tool for evaluating risk based on these influential factors.

**Table 3:** Univariate and multivariate analysis results

Variables	Univariate analysis		Multivariate analysis^a^	
	OR (95% CI)	*p*-value	OR (95% CI)	*p*-value
Age	1.01 (0.99–1.03)	0.557	1.07 (1.02–1.13)	0.005
BMI	1.03 (0.98, 1.10)	0.258	1.12 (0.96–1.31)	0.164
Pre-admission CKD	10.17 (2.78–65.62)	0.002	10.95 (0.91–296.32)	0.087
Preoperative serum creatinine	1.01 (1.01–1.02)	0.002	1.01 (1.00–1.03)	0.316
Preoperative eGFR	0.99 (0.98–1.00)	0.032	1.00 (0.98–1.02)	0.698
Preoperative cystatin C	2.54 (1.30–5.06)	0.007	2.70 (0.59–13.75)	0.211
Preoperative WBC	1.17 (1.08–1.28)	< 0.001	1.31 (1.11–1.56)	0.002
Preoperative D-dimer	1.00 (0.99, 1.01)	0.977	0.99 (0.96–1.01)	0.283
CPB duration > 180 min	4.10 (2.35–7.33)	< 0.001	3.10 (1.13, 8.96)	0.031
Drainage volume 24 h after surgery	1.00 (1.00–1.00)	0.032	1.00 (1.00–1.00)	0.758
Cleveland Clinic score	1.30 (1.15–1.46)	< 0.001	1.26 (1.02–1.58)	0.037
Urinary NGAL 0 h post-surgery	1.01 (1.01–1.02)	< 0.001	1.02 (1.01–1.03)	< 0.001
Plasma S100A8/A9 0 h post-surgery	1.35 (1.24–1.49)	< 0.001	1.33 (1.18–1.54)	< 0.001
Plasma PTX3 0 h post-surgery	1.25 (1.17–1.35)	< 0.001	1.25 (1.13–1.41)	< 0.001
Plasma CHI3L1 0 h post-surgery	1.01 (1.00–1.01)	< 0.001	1.01 (1.00–1.01)	< 0.001

^a^Adjustment was made for various categories including demographics (such as age and BMI), comorbidities (like pre-admission CKD status), baseline renal function (eGFR, serum creatinine, and cystatin C), laboratory values (for example, WBC and D-dimer), intraoperative variables (such as time of CPB), and postoperative risk factors (including drainage volume 24 h after surgery, Cleveland Clinic score, and biomarker levels 0 h post-surgery)

*BMI* Body mass index, *CKD* Chronic kidney disease, *eGFR* estimated glomerular filtration rate, *WBC* White blood cell, *CPB* Cardiopulmonary bypass, *NGAL* Neutrophil gelatinase-associated lipocalin, *PTX3* Pentraxin 3, *CHI3L1* Chitinase 3-like 1, *OR* Odds ratio, *CI* Confidence interval

**Fig. 6:**
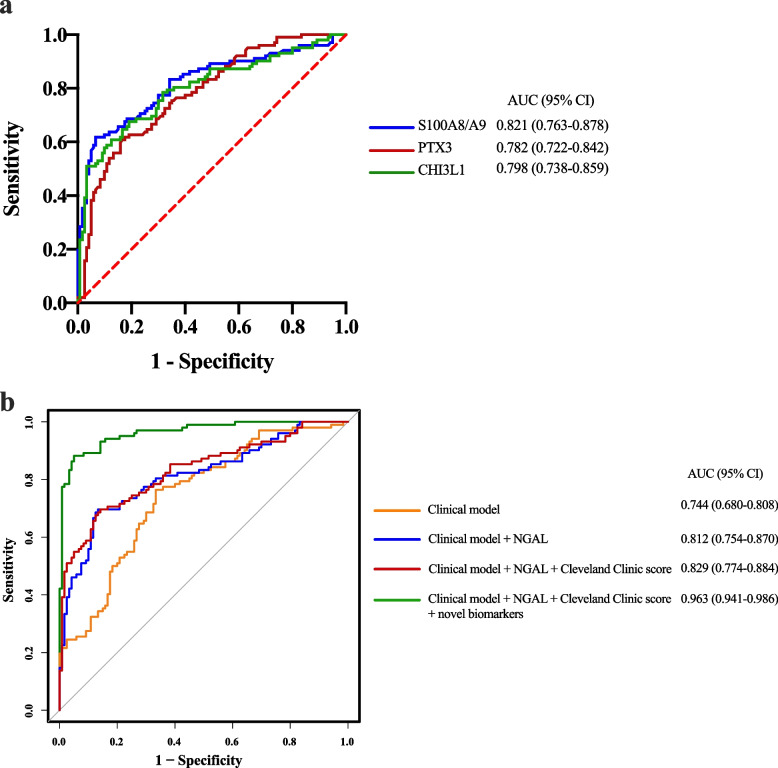
Diagnostic power of the biomarkers and different models for ASA-AKI. **a** ROC curves of plasma S100A8/A9, PTX3, and CHI3L1 levels in the model construction cohort. **b** ROC curves depict the performance of the clinical model with the inclusion or exclusion of the Cleveland Clinic score, urinary NGAL, or plasma levels of novel biomarkers (S100A8/A9, PTX3, and CHI3L1) at 0 h postoperatively for predicting ASA-AKI. PTX3, pentraxin 3; CHI3L1, chitinase 3-like 1; ASA-AKI, acute type A aortic dissection surgery-associated acute kidney injury; ROC, receiver operating characteristic; NGAL: neutrophil gelatinase-associated lipocalin; AUC, area under the curve; CI, confidence interval

In the model construction cohort, AUCs for S100A8/A9, PTX3, and CHI3L1 at 0 h after surgery were 0.821, 0.782, and 0.798, respectively (Fig. 6a), consistent with validation cohort results. When comparing various combinations of the clinical model with urinary NGAL, the Cleveland Clinic score, or novel biomarkers (S100A8/A9, PTX3, and CHI3L1), we noted that the highest AUC (0.963 [95% CI: 0.941–0.986]) was achieved with the integration of the clinical model, NGAL, Cleveland Clinic score, and novel biomarkers (Fig. 6b). Furthermore, the predominant influence on this effect was attributed to the scores derived from the novel biomarkers. The corresponding AUC in the precision-recall curve was also 0.963 (Additional file 1: Fig. S4). Calibration analysis demonstrated congruence between predicted and actual ASA-AKI occurrences (Additional file 1: Fig. S5). Decision curve analysis further validated the nomogram’s net benefit (Additional file 1: Fig. S6), while the clinical impact curve displayed effective stratification of high-risk probability (Additional file 1: Fig. S7). Notably, the prognostic model's net benefit surpassed the treat-all-patients and treat-none strategies.

## Discussion

While previous investigations have highlighted certain biomarkers for ASA-AKI, such as receptor-interacting protein kinase 3 and cystatin C, their predictive capacities remain limited [12, 20]. Moreover, it has been well-established that the occurrence of AKI can lead to CKD [21]. Given the severe consequences for patients experiencing ASA-AKI, developing a novel predictive method for timely precautions is crucial. In this study, we identified S100A8/A9, PTX3, and CHI3L1 as potential ASA-AKI biomarkers through proteomic analysis. Our findings demonstrated elevated plasma levels of S100A8/A9, PTX3, and CHI3L1 in ASA-AKI patients, confirmed across both the validation and model construction cohorts. Moreover, increased early postoperative plasma levels of these proteins independently correlated with a heightened risk of ASA-AKI development. To further enhance predictive accuracy, we constructed a nomogram based on these biomarkers and achieved promising results.

Our study highlighted elevated preoperative plasma levels of S100A8/A9, PTX3, and CHI3L1 in ATAAD patients compared to those undergoing other CPB surgeries and healthy controls. These levels further escalated postoperatively in ASA-AKI patients. Notably, prolonged CPB duration was observed in the ASA-AKI group versus the non-ASA-AKI group. We speculate that extended extracorporeal circulation might trigger kidney necroptosis, resulting in heightened plasma expression of these biomarkers. Consequently, postoperative S100A8/A9, PTX3, and CHI3L1 levels could potentially serve as novel indicators for identifying ASA-AKI risk.

Our data underscored an immediate rise in plasma S100A8/A9 levels following CPB surgery, demonstrating strong prognostic value for AKI prediction. S100A8/A9 is a leukocyte calgranulin protein that accounts for 40% of the total cytosolic protein content of neutrophils and is found at lower levels (5%) in monocytes [15]. As a calcium and arachidonic acid-binding protein, S100A8/A9 has both intracellular and extracellular functions. It is normally released through nonclassical secretion and/or during necrosis and acts as a neutrophil chemoattractant and activator. It has been discovered that S100A8/A9 is a highly sensitive marker for acute inflammation [22]. Prior studies have reported that the S100A8/A9 gene expression was increased in circulating leukocytes of patients who had undergone CPB [23]. Another study proved that conditional ablation of the S100A8 gene in myeloid cells or general knockout of the S100A9 gene in mice could ameliorate glomerulonephritis and obstructive nephropathy phenotypes due to inflammatory response inhibition [24]. The plasma S100A8/A9 level had been proven to be a useful marker for inflammation in patients with end-stage renal disease and on chronical diagnosis [25]. Notably, a recent study showed that the S100A8/A9-TLR4-NLRP3 inflammasome pathway is involved in contrast-induced AKI [26]. Our preliminary study suggests that the levels of S100A8/A9, measured immediately after the operation, might serve as a valuable early indicator for predicting the likelihood of developing ASA-AKI.

Among all soluble pattern recognition molecules, the pentraxin family plays an important role in regulating inflammation. C-reactive protein (CRP) is a member of the pentraxin family and has been widely used in clinics to measure inflammatory responses. In comparison to CRP, PTX3 is a multimeric protein that can be synthesized by various cells and released during the acute phase of inflammation, proving to be more sensitive in reflecting systemic inflammation [27]. Circulating PTX3 levels have been observed as a sensitive biomarker for risk stratification in conditions such as acute myocardial infarction, heart failure, renal injury, and acute lung injury [28–30]. However, most studies have focused on internal medicine patients, and specific data on its application in cardiac surgery involving CPB is lacking. In our current study, we measured the concentrations of PTX3 and discovered that plasma PTX3 levels might serve as a novel biomarker for predicting ASA-AKI.

CHI3L1 is a secreted glycoprotein originally isolated from human osteosarcoma cells [31]. CHI3L1 is approximately 40 kDa in size and is expressed and secreted by various cell types such as neutrophils, macrophages, vascular smooth muscle cells, and chondrocytes. Increased systemic expression of CHI3L1 has been known to be independently associated with the presence of coronary artery disease and can be used as a surrogate to measure disease progression [32]. In addition, increased systemic expression of CHI3L1 in patients with type 1 and/or type 2 diabetes mellitus was associated with progressing vascular damage in kidneys, as assessed by the level of albuminuria [32]. We hypothesize that mild inflammation and endothelial dysfunction can cause micro- and macrovascular complications that induce systemic secretion of CHI3L1. The systemic level of CHI3L1 is increased during acute inflammation as well [33, 34]. ATAAD surgery and the use of CPB can induce systemic inflammatory response syndrome that leads to ASA-AKI and the secretion of CHI3L1 might increase during this process. Several studies have linked CHI3L1 to AKI following cardiac surgery, but its diagnostic effectiveness was considered inadequate [35–37]. CHI3L1 is established as an indicator of acute inflammation in systemic conditions [33]. In our cohort, patients undergoing aortic dissection repair were all in an acute state, differing from the predominantly elective nature of typical cardiac surgeries. Additionally, the duration of CPB in ATAAD surgery significantly surpassed that of standard cardiac procedures. These factors could lead to a more intense systemic inflammatory response post-ATAAD surgery compared to standard cardiac surgery, potentially explaining the robust predictive ability of CHI3L1 in ASA-AKI.

In addition, our results showed that advanced age was associated with the occurrence of ASA-AKI. The immune function in elderly patients is generally declined and a larger portion of patients with advanced age are accompanied by various metabolic diseases, such as hypertension, hyperlipidemia, and diabetes. Prolonged CPB duration (> 180 min) was identified as another risk factor for AKI which was consistent with previous studies [3]. Renal medullary ischemia and reperfusion injury may be the most important pathophysiological change in ASA-AKI and reduced ischemia time may result in better renal and survival outcomes [38]. In contrast with previous studies [34], our study found that increased sCr level was not associated with worse kidney function. We believe that this discrepancy is due to the fact that sCr is not always a perfect surrogate to measure renal function [39] and increased creatinine may occur at a later stage of AKI. A history of CKD is recognized as a risk factor for AKI after cardiac surgery [6]. However, our study's multivariate analysis did not demonstrate an association between pre-admission CKD status and the incidence of AKI. These discrepancies could be attributed to the relatively small number of CKD cases within our patient cohort, which may have limited the statistical power to analyze risk factors.

## Limitations

Limitations of our study include the absence of standardized treatment protocols among anesthetists, perfusionists, or cardiac surgeons, potentially influencing outcomes. Surgical variations and anesthesia agents can provoke distinct inflammatory responses, warranting consideration. Additionally, mechanistic insights explaining the link between plasma S100A8/A9, PTX3, CHI3L1, and ASA-AKI remain absent. Lastly, our single-center study may not be entirely representative, necessitating multicenter investigations with rigorous protocols to validate the novel nomogram's utility.

## Conclusions

In conclusion, our findings suggested that plasma levels of S100A8/A9, PTX3, and CHI3L1 rapidly raised postoperatively in ASA-AKI patients. Moreover, the nomogram we constructed using these biomarkers demonstrated remarkable predictive capability for ASA-AKI occurrence, thus holding potential for improved clinical management.

### Supplementary Information

**Additional file 1: **Contains materials used throughout the study. **Table S1.** Demographic and clinical characteristics of the validation cohort. **Table S2.** Clinical characteristics of patients with ATAAD, CPB, and healthy control subjects at the time of enrollment. **Table S3.** Demographic and clinical characteristics of the training group and the nomogram validation group. **Table S4.** Measures of model fit variability. **Fig. S1.** Characteristics of urinary NGAL levels and their predictive values for ASA-AKI. **Fig. S2.** Characteristics of urinary S100A8/A9, PTX3, and CHI3L1 levels and their predictive values for ASA-AKI. **Fig. S3.** Correlations between plasma S100A8/A9, PTX3, and CHI3L1 levels with urinary NGAL levels and Cleveland Clinic scores. **Fig. S4.** Precision-recall curve of the nomogram in the nomogram validation cohort. **Fig. S5.** Calibration curve of the nomogram in the nomogram validation cohort. **Fig. S6.** The DCA for the prediction model. **Fig. S7.** Clinical impact curve to predict the number of patients who may develop ASA-AKI for a population size of 1000.

## Data Availability

Data sharing in the current study isavailable from the corresponding author on reasonable request.

## Abbreviation

AKI: Acute kidney injury
ASA-AKI: Acute type A aortic dissection surgery-associated acute kidney injury
ATAAD: Acute type A aortic dissection
AUC: Areas under the curve
BMI: Body mass index
CHI3L1: Chitinase 3-like 1
CI: Confidence interval
CPB: Cardiopulmonary bypass
ELISA: Enzyme-linked immunosorbent assay
KDIGO: Kidney Disease Improving Global Outcomes
NGAL: Neutrophil gelatinase-associated lipocalin
OR: Odds ratio
PTX3: Pentraxin 3
ROC: Receiver operating characteristic
SCr: Serum creatinine
