# Supplementary material for: Prediction of acute kidney injury incidence following acute type A aortic dissection surgery with novel biomarkers: a prospective observational study
Source: BMC Med. 2023 Dec 18;21:503. doi: 10.1186/s12916-023-03215-9 (PMC10729328; doi:10.1186/s12916-023-03215-9)
Supplement: Supplementary file 1 — Additional file 1: Contains materials used throughout the study. Table S1. Demographic and clinical characteristics of the validation cohort. Table S2. Clinical characteristics of patients with ATAAD, CPB, and healthy control subjects at the time of enrollment. Table S3. Demographic and clinical characteristics of the training group and the nomogram validation group. Table S4. Measures of model fit variability. Fig. S1. Characteristics of urinary NGAL levels and their predictive values for ASA-AKI. Fig. S2. Characteristics of urinary S100A8/A9, PTX3, and CHI3L1 levels and their predictive values for ASA-AKI. Fig. S3. Correlations between plasma S100A8/A9, PTX3, and CHI3L1 levels with urinary NGAL levels and Cleveland Clinic scores. Fig. S4. Precision-recall curve of the nomogram in the nomogram validation cohort. Fig. S5. Calibration curve of the nomogram in the nomogram validation cohort. Fig. S6. The DCA for the prediction model. Fig. S7. Clinical impact curve to predict the number of patients who may develop ASA-AKI for a population size of 1000. [file 12916_2023_3215_MOESM1_ESM.docx]

**Additional file 1: Fig. S1 Characteristics of urinary NGAL levels and their predictive values for ASA-AKI. a** Dynamic change of urinary NGAL levels at preoperative, 0 h, 24 h, and 48 h after ATAAD surgery. **b** ROC curves of urinary NGAL for detecting ASA-AKI at different time points. NGAL, neutrophil gelatinase-associated lipocalin; ASA-AKI, acute type A aortic dissection surgery associated acute kidney injury; ATAAD, acute type A aortic dissection; ROC, receiver operating characteristic; AUC, area under the curve; CI, confidence interval. Data were represented as mean ± SD, ***p* < 0.01.

**Additional file 1: Fig. S2** **Characteristics of urinary S100A8/A9, PTX3, and CHI3L1 levels and their predictive values for ASA-AKI.** **a-c** Correlations between plasma S100A8/A9 (**a**), PTX3 (**b**), and CHI3L1 (**c**) levels and urinary S100A8/A9, PTX3, and CHI3L1 levels at 0 h after ATAAD surgery. **d-f** Dynamic changes of urinary S100A8/A9 (**d**), PTX3 (**e**), and CHI3L1 (**f**) levels at 0 h, 24 h, and 48 h after ATAAD surgery, respectively. **g-i** ROC curves of urinary S100A8/A9 (**g**), PTX3 (**h**), and CHI3L1 (**i**) for detecting ASA-AKI at different time points. ASA-AKI, acute type A aortic dissection surgery associated acute kidney injury; PTX3, pentraxin 3; CHI3L1, chitinase 3-like 1; ROC, receiver operating characteristic; AUC, area under the curve; CI, confidence interval. Data were represented as mean ± SD, **p* < 0.05; ***p* < 0.01; ****p* < 0.001.

**Additional file 1: Fig. S3 Correlations between plasma S100A8/A9, PTX3, and CHI3L1 levels with urinary NGAL levels and Cleveland Clinic scores**. **a-c** Correlations between plasma S100A8/A9 (**a**), PTX3 (**b**), and CHI3L1 (**c**) levels and urinary NGAL levels at 0 h after ATAAD surgery. **d-f** Correlations between plasma S100A8/A9 (**d**), PTX3 (**e**), and CHI3L1 (**f**) levels at 0 h after ATAAD surgery and Cleveland Clinic scores. **g** Comparison of ROC curves of Cleveland Clinic score, urinary NGAL, S100A8/A9, PTX3, and CHI3L1 at 0 h after surgery for diagnosing ASA-AKI. PTX3, pentraxin 3; CHI3L1, chitinase 3-like 1; NGAL, neutrophil gelatinase-associated lipocalin; ROC, receiver operating characteristic; ATAAD, acute type A aortic dissection; ASA-AKI, acute type A aortic dissection surgery associated acute kidney injury; AUC, area under the curve; CI, confidence interval.

**Additional file 1: Fig. S4** Precision-recall curve of the nomogram in the nomogram validation cohort. AUC, area under the curve.


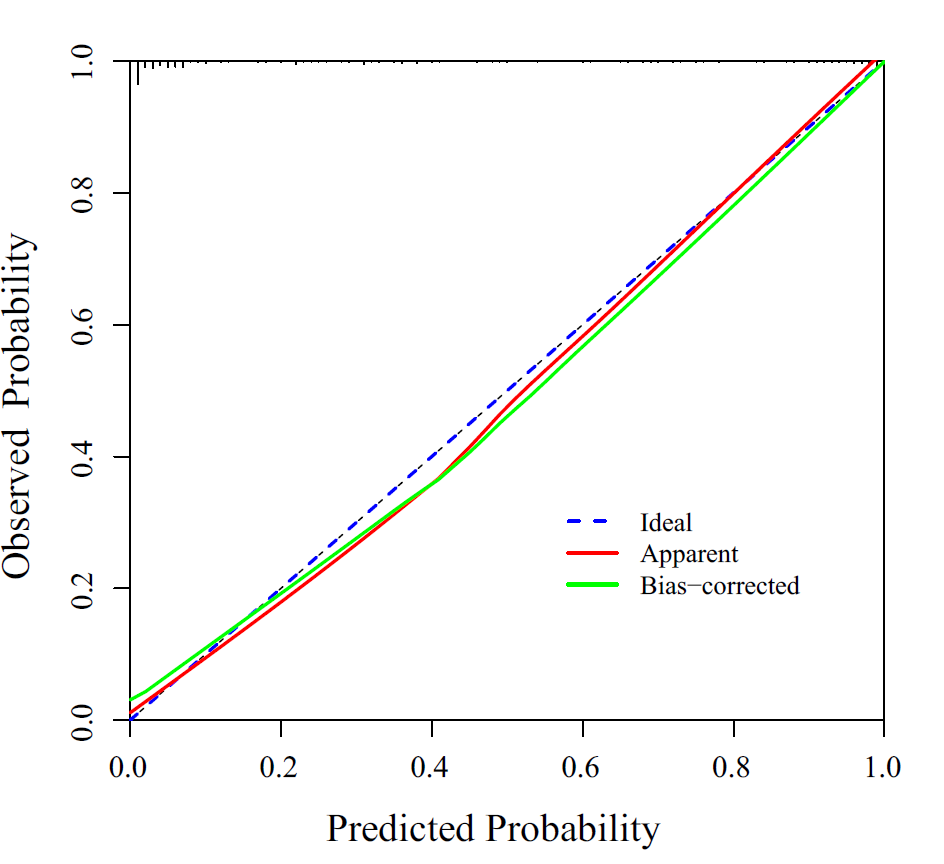


**Additional file 1: Fig. S5 Calibration curve of the nomogram in the nomogram validation cohort.** The calibration curve illustrates the agreement between predicted risks (X-axis) and actual outcomes (Y-axis) for the nomogram.

**Additional file 1: Fig. S6 The DCA for the prediction model.** The DCA demonstrates that the nomogram enhances net benefits and encompasses a wide range of threshold probabilities in predicting the occurrence of ASA-AKI. DCA, decision curve analysis; ASA-AKI, acute type A aortic dissection surgery associated acute kidney injury.


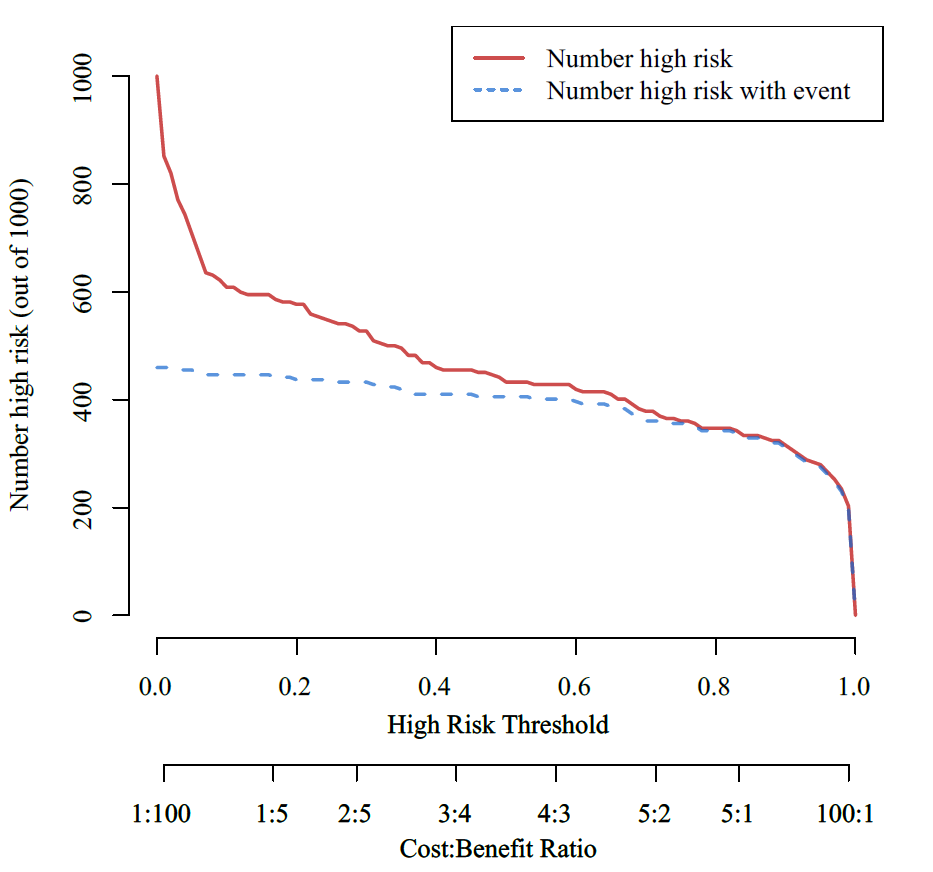


**Additional file 1: Fig. S7 Clinical impact curve to predict the number of patients who may develop ASA-AKI for a population size of 1000.** The red curve denotes the predicted number of ASA-AKI cases at varying threshold probabilities, while the blue curve represents the actual number of ASA-AKI cases. ASA-AKI, acute type A aortic dissection surgery associated acute kidney injury.

**Additional file 1: Table S1** Demographic and clinical characteristics of the validation cohort.

| Variables | ASA-AKI  (n = 37) | Non-ASA-AKI  (n = 39) | *p*-value |
| --- | --- | --- | --- |
| ***Preoperative parameters*** |  |  |  |
| Demographics |  |  |  |
| Age (year) | 53.1 ± 13.7 | 56.1 ± 12.3 | 0.322 |
| Male (%) | 27 (73.0) | 29 (74.4) | 0.891 |
| BMI (kg/m2) | 25.6 ± 4.3 | 25.4 ± 3.3 | 0.800 |
| Medical history |  |  |  |
| Hypertension (%) | 27 (73.0) | 29 (74.4) | 0.891 |
| Diabetes mellitus (%) | 2 (5.4) | 1 (2.6) | 0.610 |
| Previous cardiac surgery (%) | 3 (8.1) | 1 (2.6) | 0.352 |
| Cerebrovascular disease (%) | 4 (10.8) | 4 (10.3) | 1.000 |
| CKD (%) | 5 (13.5) | 2 (5.1) | 0.256 |
| CKD stage 1 (%) | 2 (5.4) | 1 (2.6) | 0.610 |
| CKD stage 2 (%) | 3 (8.1) | 1 (2.6) | 0.352 |
| Involving renal artery (%) | 23 (62.2) | 26 (66.7) | 0.682 |
| Limb ischemia (%) | 0 (0) | 1 (2.6) | 1.000 |
| Mesenteric ischemia (%) | 1 (2.7) | 1 (2.6) | 1.000 |
| Cerebral ischemia (%) | 1 (2.7) | 2 (5.1) | 1.000 |
| Coronary ischemia (%) | 2 (5.4) | 1 (2.6) | 0.610 |
| Hypotension (%) | 1 (2.7) | 1 (2.6) | 1.000 |
| Preoperative laboratory results |  |  |  |
| WBC (10^9^/L) | 11.0 ± 3.8 | 9.7 ± 3.5 | 0.135 |
| Hemoglobin (g/L) | 126.3 ± 25.1 | 127.3 ± 18.5 | 0.852 |
| Creatinine (μmol/L) | 74.0 ± 20.1 | 69.1 ± 18.1 | 0.268 |
| BUN (mmol/L) | 6.2 ± 2.0 | 6.2 ± 1.9 | 0.902 |
| Cystatin C (mg/L) | 1.0 ± 0.5 | 0.9 ± 0.5 | 0.509 |
| eGFR (ml/min) | 104.8 ± 36.8 | 110.3 ± 24.9 | 0.455 |
| Albuminuria (%) | 3 (8.1) | 0 (0) | 0.111 |
| D-Dimer (ng/mL) | 7.3 (4.1, 33.1) | 4.5 (1.8, 12.8) | 0.234 |
| ***Intraoperative parameters*** |  |  |  |
| CPB duration (min) | 193.8 ± 33.8 | 175.3 ± 42.2 | 0.039 |
| Cross-clamp duration (min) | 144.6 ± 30.4 | 128.0 ± 37.4 | 0.038 |
| Hypothermia circulation arrest time (min) | 24.0 ± 11.6 | 23.4 ± 6.2 | 0.885 |
| ***Postoperative parameters*** |  |  |  |
| Drainage volume 24 h after surgery (ml) | 700.0 (500, 900) | 410.0 (270.0, 580.0) | 0.048 |
| Dialysis (%) | 6 (16.2) | 0 (0) | 0.011 |
| Cleveland Clinic score | 5.0 ± 1.6 | 3.4 ± 1.3 | < 0.001 |
| Mechanical ventilation time (h) | 33.5 (13.0, 79.8) | 13.0 (7.0, 18.5) | 0.016 |
| In-hospital death (%) | 9 (24.3) | 3 (7.7) | 0.047 |
| ICU stay (d) | 5.0 (3.0, 8.0) | 3.0 (2.0, 4.0) | 0.001 |
| Hospital stay (d) | 16.5 (12.3, 22.5) | 13.0 (11.0, 17.0) | 0.038 |

BMI, body mass index; WBC, white blood cell; BUN, blood urea nitrogen; eGFR, estimated glomerular filtration rate; CKD, chronic kidney disease; CPB, cardiopulmonary bypass; ICU, intensive care unit. ASA-AKI, acute type A aortic dissection surgery associated acute kidney injury.

**Additional file 1: Table S2** Clinical characteristics of patients with ATAAD, CPB surgery, and healthy control subjects at the time of enrollment.

| Variables | ATAAD  (n = 76) | CPB surgery  (n = 52) | HC  (n = 37) |
| --- | --- | --- | --- |
| ***Preoperative parameters*** |  |  |  |
| Demographics |  |  |  |
| Age (year) | 54.6 ± 13.0 | 55.6 ± 11.5 | 55.8 ± 8.2 |
| Male (%) | 56 (73.7) | 38 (73.1) | 27 (73.0) |
| BMI (kg/m2) | 25.5 ± 3.8 | 24.9 ± 2.9 | 24.8 ± 2.1 |
| Medical history |  |  |  |
| Hypertension (%) | 56 (73.7) | 36 (69.2) | 26 (70.3) |
| Diabetes mellitus (%) | 3 (3.9) | 3 (5.8) | 2 (5.4) |
| Previous cardiac surgery (%) | 4 (5.3) | 3 (5.8) | 1 (2.7) |
| Cerebrovascular disease (%) | 8 (10.5) | 6 (11.5) | 3 (8.1) |
| CKD (%) | 7 (9.2) | 4 (7.7) | 0 (0) |
| CKD stage 1 (%) | 3 (3.9) | 2 (3.8) | 0 (0) |
| CKD stage 2 (%) | 4 (5.3) | 2 (3.8) | 0 (0) |
| Involving renal artery (%) | 49 (64.5) | NA | NA |
| Limb ischemia (%) | 1 (1.3) | NA | NA |
| Mesenteric ischemia (%) | 2 (2.6) | NA | NA |
| Cerebral ischemia (%) | 3 (3.9) | NA | NA |
| Coronary ischemia (%) | 3 (3.9) | NA | NA |
| Hypotension (%) | 2 (2.6) | 1 (1.9) | NA |
| Preoperative laboratory results |  |  |  |
| WBC (10^9^/L) | 10.4 ± 3.7 | 9.0 ± 2.1 | 7.6 ± 1.3 |
| Hemoglobin (g/L) | 126.8 ± 22.0 | 125.7 ± 10.5 | 128.3 ± 10.7 |
| Creatinine (μmol/L) | 71.5 ± 19.2 | 71.2 ± 13.1 | 62.1 ± 6.2 |
| BUN (mmol/L) | 6.2 ± 2.0 | 5.8 ± 1.2 | 5.3 ± 1.5 |
| Cystatin C (mg/L) | 0.9 ± 0.5 | 0.8 ± 0.4 | NA |
| eGFR (ml/min) | 107.6 ± 31.2 | 108.7 ± 13.6 | 115.3 ± 13.0 |
| Albuminuria (%) | 3 (3.9) | 1 (1.9) | 0 (0) |
| D-Dimer (ng/mL) | 5.1 (2.5, 13.6) | 5.4 (3.0, 6.8) | NA |
| ***Intraoperative parameters*** |  |  |  |
| CPB duration (min) | 184.3 ± 39.2 | 110.6 ± 13.9 | NA |
| Cross-clamp time (min) | 136.1 ± 35.0 | 73.1 ± 8.5 | NA |
| Hypothermia circulation arrest time (min) | 23.8 ± 9.2 | NA | NA |
| ***Postoperative parameters*** |  |  |  |
| Drainage volume 24 h after surgery (ml) | 530 (307.5, 800.0) | 290.0 (185.0, 420.0) | NA |
| Dialysis (%) | 6 (7.9) | 1 (1.9) | NA |
| AKI (%) | 37 (48.7) | 5 (9.6) | NA |
| Cleveland Clinic score | 4.1 ± 1.6 | 3.4 ± 1.1 | NA |
| Mechanical ventilation time (h) | 16.0 (8.0, 45.0) | 6.0 (5.0, 8.0) | NA |
| In-hospital death (%) | 12 (15.8) | 1 (1.9) | NA |
| ICU stay (d) | 3.5 (2.3, 6.5) | 3.0 (2.0, 4.0) | NA |
| Hospital stay (d) | 14.0 (11.0, 18.0) | 12.0 (10.0, 15.0) | NA |

BMI, body mass index; WBC, white blood cell; BUN, blood urea nitrogen; eGFR, estimated glomerular filtration rate; CKD, chronic kidney disease; AKI, acute kidney injury; ICU, intensive care unit; ATAAD, acute type A aortic dissection; CPB, cardiopulmonary bypass; HC, healthy control.

**Additional file 1: Table S3** Demographic and clinical characteristics of the training group and the nomogram validation group.

| Variables | Training group  （n = 155） | Nomogram validation group  （n = 67） | *p*-value |
| --- | --- | --- | --- |
| ***Preoperative parameters*** |  |  |  |
| Demographics |  |  |  |
| Age (year) | 53.2 ± 13.5 | 52.7 ± 13.7 | 0.816 |
| Male (%) | 119 (76.8) | 53 (79.1) | 0.703 |
| BMI (kg/m2) | 26.2 ± 4.6 | 26.3 ± 4.6 | 0.924 |
| Medical history |  |  |  |
| Hypertension (%) | 123 (79.4) | 55 (82.1) | 0.639 |
| Diabetes mellitus (%) | 2 (1.3) | 6 (9.0) | 0.010 |
| Previous cardiac surgery (%) | 7 (4.5) | 3 (4.5) | 1.000 |
| Cerebrovascular disease (%) | 15 (9.7) | 10 (14.9) | 0.256 |
| CKD (%) | 14 (9.0) | 3 (4.5) | 0.241 |
| CKD stage 1 (%) | 5 (3.2) | 0 (0) | 0.193 |
| CKD stage 2 (%) | 6 (3.9) | 3 (4.5) | 1.000 |
| CKD stage 3 (%) | 3 (1.9) | 0 (0) | 0.555 |
| Involving renal artery (%) | 102 (65.8) | 48 (71.6) | 0.394 |
| Limb ischemia (%) | 13 (8.4) | 3 (4.5) | 0.403 |
| Mesenteric ischemia (%) | 4 (2.6) | 3 (4.5) | 0.678 |
| Cerebral ischemia (%) | 9 (5.8) | 4 (6.0) | 1.000 |
| Coronary ischemia (%) | 6 (3.9) | 3 (4.5) | 1.000 |
| Hypotension (%) | 2 (1.3) | 3 (4.5) | 0.326 |
| Preoperative laboratory results |  |  |  |
| WBC (10^9^/L) | 11.9 ± 4.2 | 12.2 ± 3.3 | 0.716 |
| Hemoglobin (g/L) | 128.8 ± 20.8 | 132.2 ± 18.2 | 0.247 |
| Creatinine (μmol/L) | 95.1 ± 54.9 | 89.4 ± 32.3 | 0.318 |
| BUN (mmol/L) | 7.2 ± 3.0 | 6.8 ± 2.1 | 0.223 |
| Cystatin C (mg/L) | 1.0 ± 0.4 | 0.9 ± 0.4 | 0.211 |
| eGFR (ml/min) | 95.0 ± 37.9 | 95.4 ± 33.0 | 0.953 |
| Albuminuria (%) | 7 (4.5) | 1 (1.5) | 0.440 |
| D-Dimer (ng/mL) | 7.2 (3.7, 14.9) | 6.3 (4.2, 14.4) | 0.677 |
| ***Intraoperative parameters*** |  |  |  |
| CPB duration (min) | 191.5 (161.0, 224.0) | 177.0 (157.3, 222.8) | 0.642 |
| Cross-clamp time (min) | 137.0 (115.5, 170.0) | 134.0 (112.0, 175.8) | 0.796 |
| Hypothermia circulation arrest time (min) | 26.0 (18.0, 32.3) | 25.0 (21.3, 31.0) | 0.351 |
| ***Postoperative parameters*** |  |  |  |
| Drainage volume 24 h after surgery (ml) | 420.0 (270.0, 645.0) | 450.0 (300.0, 600.0) | 0.532 |
| Dialysis (%) | 11 (7.1) | 7 (10.4) | 0.401 |
| AKI (%) | 71 (45.8) | 31 (46.3) | 0.949 |
| Cleveland Clinic score | 5.6 ± 2.4 | 5.2 ± 2.7 | 0.268 |
| Mechanical ventilation time (h) | 21.5 (14.5, 70.0) | 20.0 (13.5, 98.0) | 0.525 |
| In-hospital death (%) | 14 (9.0) | 5 (7.5) | 0.701 |
| ICU stay (d) | 5.0 (3.0, 7.0) | 5.0 (3.0, 10.0) | 0.759 |
| Hospital stay (d) | 18.0 ± 9.2 | 19.9 ± 12.8 | 0.252 |

BMI, body mass index; WBC, white blood cell; BUN, blood urea nitrogen; eGFR, estimated glomerular filtration rate; CKD, chronic kidney disease; CPB, cardiopulmonary bypass; ICU, intensive care unit.

**Additional file 1: Table S4** Measures of model fit variability.

| Model | Deviance | AIC | BIC | McFadden’s R^2^ | Cox & Snell’s R^2^ | Nagelkerke’s R^2^ |
| --- | --- | --- | --- | --- | --- | --- |
| Forward | 100.168 | 121.294 | 152.955 | 0.664 | 0.601 | 0.802 |
| Backward | 104.868 | 122.868 | 153.492 | 0.658 | 0.596 | 0.797 |
| Both | 101.294 | 122.168 | 155.139 | 0.664 | 0.600 | 0.802 |

AIC, Akaike information criterion; BIC, Bayesian information criterion.
